# Genome-Wide Survey of Large Rare Copy Number Variants in Alzheimer’s Disease Among Caribbean Hispanics

**DOI:** 10.1534/g3.111.000869

**Published:** 2012-01-01

**Authors:** Mahdi Ghani, Dalila Pinto, Joseph H. Lee, Yakov Grinberg, Christine Sato, Danielle Moreno, Stephen W. Scherer, Richard Mayeux, Peter St. George-Hyslop, Ekaterina Rogaeva

**Affiliations:** * Tanz Centre for Research in Neurodegenerative Diseases, University of Toronto, Toronto, Ontario M5S 3H2, Canada; ‡‡ Department of Medicine, University of Toronto, Toronto, Ontario M5S 1A8, Canada; † Genetics and Genomic Biology, Hospital for Sick Children, Toronto, Ontario M5G 1L7, Canada; ‡ Taub Institute for Research on Alzheimer’s Disease and the Aging Brain and; § Gertrude H. Sergievsky Center, Columbia University Medical Center, New York, New York 10032; ** Departments of Neurology, Psychiatry, and Medicine, College of Physicians and Surgeons, and; †† Department of Epidemiology, School of Public Health, Columbia University, New York, New York 10032, and; §§ Cambridge Institute for Medical Research; *** Department of Clinical Neurosciences, University of Cambridge, Cambridge CB2 0XY, United Kingdom

**Keywords:** gene, deletion, duplication, Alzheimer’s Disease, copy number variants

## Abstract

Recently genome-wide association studies have identified significant association between Alzheimer’s disease (AD) and variations in *CLU*, *PICALM*, *BIN1*, *CR1*, *MS4A4*/*MS4A6E*, *CD2AP*, *CD33*, *EPHA1*, and *ABCA7*. However, the pathogenic variants in these loci have not yet been found. We conducted a genome-wide scan for large copy number variation (CNV) in a dataset of Caribbean Hispanic origin (554 controls and 559 AD cases that were previously investigated in a SNP-based genome-wide association study using Illumina HumanHap 650Y platform). We ran four CNV calling algorithms to obtain high-confidence calls for large CNVs (>100 kb) that were detected by at least two algorithms. Global burden analyses did not reveal significant differences between cases and controls in CNV rate, distribution of deletions or duplications, total or average CNV size; or number of genes affected by CNVs. However, we observed a nominal association between AD and a ∼470 kb duplication on chromosome 15q11.2 (*P* = 0.037). This duplication, encompassing up to five genes (*TUBGCP5*, *CYFIP1*, *NIPA2*, *NIPA1*, and *WHAMML1*) was present in 10 cases (2.6%) and 3 controls (0.8%). The dosage increase of *CYFIP1* and *NIPA1* genes was further confirmed by quantitative PCR. The current study did not detect CNVs that affect novel AD loci identified by recent genome-wide association studies. However, because the array technology used in our study has limitations in detecting small CNVs, future studies must carefully assess novel AD genes for the presence of disease-related CNVs.

Alzheimer’s disease (AD) is the most common form of dementia, affecting ∼30% of individuals over 80 years of age (Mayeux 2003). The hallmark of AD brain pathology is characterized by the accumulation of a neurotoxic proteolytic derivative of the amyloid precursor protein (APP; Aβ peptides) and the formation of intraneuronal tau-associated neurofibrillary tangles. The majority of AD cases are sporadic (∼95%), with onset after 65 years of age. One half of the genetic variance is attributable to mutations in *APP*, *PSEN1*, *PSEN2*, and the *APOE* e4-allele, and they have been shown to cause the overproduction or reduced clearance of Aβ (Rogaeva *et al*. 2006). More recently, it was demonstrated that single nucleotide polymorphisms (SNP) in *SORL1* are significantly associated with late-onset AD in several independent cohorts (Rogaeva *et al*. 2007; Reitz *et al*. 2011). In addition, genome-wide association studies (GWAS) of large case-control datasets have identified significant association between late-onset AD and SNPs in *CLU*, *PICALM*, *BIN1*, *CR1*, *MS4A4*/*MS4A6E*, *CD2AP*, *CD33*, *EPHA1*, and *ABCA7* (Harold *et al*. 2009; Lambert *et al*. 2009; Carrasquillo *et al*. 2010; Hollingworth *et al*. 2011; Naj *et al*. 2011). Our recent GWAS of a Caribbean Hispanic cohort has supported the association with SNPs in the *CLU*, *PICALM*, and *BIN1* genes (Lee JH *et al*. 2010). However, the pathogenic variants in these novel AD loci have not yet been found.

Copy number variants (CNV) have been associated with several neuropsychiatric disorders, such as autism, schizophrenia, and bipolar disorder (Cook and Scherer 2008; Lee and Scherer 2010). Furthermore, rare duplications of the APP locus are associated with dominant early-onset AD, which support the possibility of the existence of disease-related CNVs in other AD genes (McNaughton *et al*. 2010). In fact, recently Brouwers *et al.* proposed that the association between *CR1* and AD might be explained by intragenic CNVs that translate into two major CR1 isoforms (Brouwers *et al*. 2011). To date, there are only two published genome-wide case-control studies that assess the contribution of CNVs to AD in North American populations (Heinzen *et al*. 2010; Swaminathan *et al*. 2011). However, in both studies, CNV calls were detected using a single method (PennCNV), and no overall case-control differences were observed in the CNV rate, size, presence of rare genic CNVs, or number of genes disrupted by CNVs. The only borderline association was reported by Heinzen *et al.* for a ∼500 kb duplication at 15q13.3 affecting the *CHRNA7* gene that encodes the neuronal nicotinic cholinergic receptor (*P* = 0.053, uncorrected for multiple testing) (Heinzen *et al*. 2010).

To evaluate the contribution of rare genomic variants to risk of late-onset AD, we analyzed a Caribbean Hispanic dataset that was previously assessed in a SNP-based GWAS (Lee JH *et al*. 2010). We focused our investigation on large rare CNVs that might contribute significantly to disease risk, as was previously demonstrated in other neuropsychiatric disorders (Kirov *et al*. 2009; Zhang *et al*. 2009; Glessner *et al*. 2010). To maximize CNV discovery, we used multiple CNV detection methods.

## METHODS

### Sample collection and genotyping

The study was approved by the Institutional Review Boards of Columbia University and the University of Toronto. The Caribbean Hispanic case-control dataset, consisting of participants predominantly originating from the Dominican Republic and Puerto Rico, was described previously (Lee JH *et al*. 2010). Briefly, the dataset included 559 unrelated cases with late-onset AD and 554 unrelated controls similar in age and sex distribution. The mean (SD) age at onset of AD was 80.0 (8.0) years, and the mean (SD) age at last examination of the controls was 78.9 (6.4) years. In both the control and AD groups, 70% of the participants were women. The diagnosis of AD was based on the National Institute of Neurological Disorders and Stroke–Alzheimer's Disease and Related Disorders Association criteria (McKhann *et al*. 1984).

All DNA samples were isolated from whole blood and were randomly distributed in genotyping plates. All samples were genotyped on Illumina HumanHap 650Y arrays at the same laboratory. The dataset consisted only of samples that previously passed SNP-based quality control procedures (*e.g.* gender miscalls and relatedness checks) (Lee JH *et al*. 2010). Our preliminary analysis was done as a blind study, and the affection status of the samples was only disclosed after the CNV detection procedures were completed.

### Quality control and CNV detection

Raw intensity array data were normalized within and across samples using Illumina's BeadStudio software v.3.3.7. To maximize CNV discovery, we ran four different CNV calling algorithms, QuantiSNP (Colella *et al*. 2007), iPattern (Pinto *et al*. 2011), PennCNV (Wang *et al*. 2007), and CNVpartition (implemented in BeadStudio). To obtain high-confidence CNV calls, a stringent CNV dataset was generated by taking the CNV calls by iPattern that were also found by at least one additional algorithm (either PennCNV or QuantiSNP). Specifically, each CNV detected by two methods was merged using the outside probe boundaries (*i.e.* union of the CNVs) as described previously (Pinto *et al*. 2010), and it needed to overlap in at least 50% of its length. Previously (using Illumina 1M arrays) we showed that stringent CNVs >30 kb detected by both iPattern and QuantiSNP were confirmed by quantitative PCR (qPCR) to be true events at 95% confidence (Pinto *et al*. 2010). Here, given the lower resolution of the current 650K array, we assumed that a comparable sensitivity would be able to detect large CNVs (>100 kb). To minimize overestimation of reported boundaries, the third algorithm was only used for support. The fourth algorithm, CNV partition, was used to visualize large CNVs.

Poor quality samples were excluded from the study if they met the following criteria: chip call rate < 97%; log R ratio standard deviation > 0.27; B allele frequency standard deviation > 0.17; and PennCNV wave factor > 0.04 or ≤ 0.04 (Diskin *et al*. 2008). We excluded CNV calls when they failed stringent quality control (QC) criteria: <5 probes, <100 kb size, or low confidence QuantiSNP score (log Bayes factor < 15), as these CNVs were likely to be unreliable at the current array resolution. We also excluded CNV calls within hypervariable centromere proximal bands and those overlapping immunoglobulin regions, as both are known to be prone to artifactual CNV calling and thus false discoveries.

Finally, we removed samples that had an excessive number of CNVs detected by each algorithm (*i.e.* samples with a number of CNV calls exceeding the third quartile plus three times the interquartile range). The resulting cutoff for the number of CNVs per sample was 67 CNV calls for PennCNV, 35 calls for QuantiSNP, and 35 calls for iPattern. Chromosome X and all CNVs > 1 Mb detected by any algorithm were inspected manually. Samples with excessive aggregate length of CNVs, as well as samples with CNVs > 7.5 Mb (likely karyotyping abnormalities) were visually inspected by plotting their intensities and allelic ratios, and removed from burden analyses (supporting information, Table S1).

For the purpose of burden analysis, CNVs with more than 50% of their length overlapping segmental duplications were discarded; CNVs found in >1% of cases and controls were not considered further. A total of 392 cases (106 males, 286 females) and 357 controls (104 males, 253 females) passed all QC steps and were used in subsequent analyses. The female/male ratio and age at onset in the dataset that passed all QC steps remained similar to original dataset: ∼70% females, the mean age at onset (SD) of AD cases was 77.1 (8.5) years, and the mean age at last examination (SD) of the controls was 79.5 (6.1) years.

### CNV burden analyses

To determine whether cases show a greater genome-wide burden of rare CNVs compared with controls, CNV burden analyses were conducted using PLINK v1.07 and a permutation procedure (one-sided, 100,000 permutations) (Purcell *et al*. 2007). *P* values were estimated for the number of CNVs per individual (CNV rate), for CNV sample proportion (fraction of samples with one or more CNVs), and for the total or average size ranges of CNV calls. Genome-wide *P* values were further corrected (*Pcorr*) for potential global case-control differences in CNV rate and size. CNVs found to be enriched in AD cases compared with controls or found only in AD cases were further evaluated by comparison with the Database of Genomic Variants (DGV), a catalog of CNVs found in control subjects of diverse populations, and by comparison with 5000 Caucasian controls previously used in an autism CNV study (Pinto *et al*. 2010). However, the controls in the autism study and DGV database were not specifically screened for AD symptoms.

### CNV validation

Primers for qPCR were designed using the Primer3 software. The samples were screened for dosage aberrations using qPCR, amplifying 5 ng of DNA with SYBR Green reagent (TaKaRa Mirus Bio, Madison, WI) on an ABI7500 system (Applied Biosystems, Foster City, CA). The duplication at 15q11.2 was assessed with two sets of primers targeting the *NIPA1* and *CYFIP1* genes: (1) (NIPA1-F) 5′-tctcctgaaggaaaagctcaa and (NIPA1-R) 5′-ctcagactttggggagtgga; (2) (CYFIP1-F) 5′-aggccaaccacaacgtgtc and (CYFIP1-R) 5′-agcagtagttgggcaggaag. The beta-globin gene (HBB) was used as the endogenous control: (HBB-F) 5′-gcaacctcaaacagacacca and (HBB-R) 5′-cctcaccaccaacttcatcc. An unrelated control DNA sample without this CNV was used as a calibration sample. The relative dosage (in triplicate) was determined by the comparative threshold cycle method (ddCt) implemented in the ABI Prism sequence detection software (v.1.3.1).

## Results

### CNV characteristics

Overall, we detected 1774 stringent CNVs with sizes ≥100 kb in the 392 cases and 357 controls that passed the QC steps (mean size = 252,651 bp; median size = 176,893 bp). This stringent CNV dataset was composed of 932 CNV calls in cases (52.5%) and 842 calls in controls (47.5%). We did not observe significant differences in the number of deletions between cases (n = 397; 22.4%) and controls (n = 367; 20.7%) or in the number of duplications between cases (n = 535; 30.2%) and controls (n = 475; 26.8%). Hence, there was no significant global enrichment between cases and controls for the total number of CNV calls or for deletions or duplications. However, we observed a nominal association between AD and a ∼470 kb (20.3–20.7 Mb NCBI36/hg18) duplication on chr15q11.2 (χ^2^ = 3.206; uncorrected one-tailed *P* = 0.037). This duplication, encompassing up to five genes (*TUBGCP5*, *CYFIP1*, *NIPA2*, *NIPA1*, and *WHAMML1*) and flanked by two low-copy repeats BP1–BP2, was present in 10 cases (2.6%) and in 3 controls (0.8%). The dosage increase of the *CYFIP1* and *NIPA1* genes in AD patients was further confirmed by qPCR (Figure S1).

### Analyses of large rare CNVs

A total of 734 stringent rare CNVs ≥ 100 kb with a frequency ≤ 1% in the total sample set were observed in our dataset (mean size = 292,240 bp; median size = 200,981 bp), including 277 deletions and 457 duplications (Table 1, Table S2). Three hundred ninety (390) rare large CNVs were detected in 255 cases (65.0%), and 344 of these CNVs were found in 224 controls (62.7%) (case/control ratio = 1.03; *P* = 0.35) (Table S2). We did not detect significant differences in the distribution of large rare deletions or duplications between cases and controls (Table 1). Furthermore, no significant association with AD was found in the total size of rare CNVs (case/control ratio = 0.94; *P* = 0.77). Similarly the average size of rare CNVs was not different between cases and controls (case/control ratio = 0.88; *P* = 0.94) (Table 1).

**Table 1:** Global rare CNV burden analyses with respect to CNV size and CNV rate

				CNV Rate				CNV Sample Proportion				Total CNV Size (kb)				Average CNV Size (kb)		
Type	Classification	Total CNVs (n)		*P*	Case/ctrl ratio	Baseline rate (ctrl)		*P*	Case/ctrl ratio	Baseline rate (ctrl)		*P*	Case/ctrl ratio	Baseline rate (ctrl)		*P*	Case/ctrl ratio	Baseline rate (ctrl)
	**None**																	
All	All	734		0.3466	1.0325	0.9636		0.2800	1.0367	0.6275		0.7659	0.9391	462.8		0.9375	0.8817	306.9
Deletions	All	277		0.2581	1.0915	0.3529		0.5309	1.0003	0.3137		0.5827	0.9667	303.1		0.8215	0.8560	270.8
Duplications	All	457		0.5260	0.9985	0.6106		0.3519	1.0375	0.4426		0.7405	0.9417	441.2		0.7664	0.9363	329.6
	**CNV size**																	
All	100–500 kb	644		0.2486	1.0575	0.8347		0.1420	1.0723	0.5686		0.8439	0.9350	326.3		0.9062	0.9455	218.3
	≥ 500 kb	90		0.7717	0.8704	0.1289		0.7900	0.8673	0.1176		0.3320	1.0638	890.9		0.3650	1.0522	823.7
	≥ 1 Mb	12		0.2400	1.8223	0.0112		0.2400	1.8223	0.0112		0.7594	0.8541	2104.0		0.7594	0.8541	2104.0
Deletions only	100–500 kb	255		0.1927	1.1265	0.3193		0.3865	1.0433	0.2885		0.4137	1.0221	212.5		0.8822	0.9364	191.8
	≥ 500 kb	22		0.7978	0.7590	0.0336		0.8665	0.6831	0.0336		0.3323	1.1512	1005.0		0.4419	1.0448	1005.0
	≥ 1 Mb	5		0.5447	1.3661	0.0056		0.5447	1.3661	0.0056		0.7016	0.8399	2311.0		0.7016	0.8399	2311.0
Duplications only	100–500 kb	389		0.4605	1.0147	0.5154		0.2701	1.0636	0.3838		0.8933	0.9129	323.8		0.9263	0.9230	245.3
	≥ 500 kb	68		0.6889	0.9106	0.0952		0.6505	0.9401	0.0868		0.3689	1.0507	817.9		0.2667	1.0972	744.2
	≥ 1 Mb	7		0.2633	2.2778	0.0056		0.2633	2.2778	0.0056		0.6248	0.9014	1897.0		0.6248	0.9014	1897.0

From 392 cases and 357 controls, rare CNVs >100 kb were seen in 255 cases and 224 controls. Rare CNVs were defined as those with a frequency ≤1% in the total sample set. CNVs per individual (CNV rate), fraction of samples with one or more CNVs (CNV sample proportion), and total or average size ranges of CNV calls were compared for cases and controls.

Global burden analyses were further extended by stratifying rare CNVs according to size (*e.g.* >500 kb or >1 Mb) and CNVs with genic content. None of these strategies revealed significant differences between cases and controls (Tables 1 and 2). For instance, the case/control ratio for all genic CNVs was 0.94 (1.1 for deletions and 0.9 for duplications), and no considerable enrichment was found for CNV size in any range or excess of gene-disrupting CNVs (Tables 1 and 2).

**Table 2:** Global rare CNV burden: gene count in 392 cases *vs.* 357 controls

Type	Classification	*P*	Case/Control Ratio	Baseline Rate (Controls)	*P_corr_*
	**None**				
All	All	0.6138	0.9443	1.796	0.8109
Deletions only	All	0.3441	1.1552	0.4902	0.4745
Duplications only	All	0.8136	0.8659	1.305	0.3639
	**CNV size**				
All	100–500 kb	0.6251	0.9599	1.148	0.5227
	≥ 500 kb	0.5694	0.9186	0.6471	0.9987
	≥ 1 Mb	0.5166	0.9555	0.2857	0.5154
Deletions only	100–500 kb	0.3937	1.0928	0.2521	0.9470
	≥ 500 kb	0.2965	1.2213	0.2381	0.5682
	≥ 1 Mb	0.6019	0.8995	0.2269	0.6848
Duplications only	100–500 kb	0.6976	0.9220	0.8964	0.4880
	≥ 500 kb	0.7764	0.7423	0.409	0.4356
	≥ 1 Mb	0.4459	1.1710	0.05882	0.5912

### Candidate novel CNVs

We observed 12 CNVs >1 Mb that were detected in eight AD cases and four controls. Six CNVs were found only in AD cases and were observed neither in Hispanic controls nor in the DGV (Table S3), suggesting that they might be novel structural abnormalities with potential functional significance for AD. For example, in case NY1811 (age at onset 73), we observed a 1.9 Mb duplication on chr 2p16.3 (Figure S2A) encompassing the entire neurexin1 gene (*NRXN1*) that encodes a neuronal cell surface protein involved in cell recognition and cell adhesion. Genome-wide CNV studies previously implicated *NRXN1* deletions in autism and schizophrenia (Ching *et al*. 2010; Magri *et al*. 2010). Our study is the first report describing a duplication of *NRXN1* in an AD case.

In AD case NY1261 (age at onset 89), we detected a 1.4 Mb deletion on chromosome 17p13.1-2 and a 563 kb deletion on 3p21.31 (Figure S2B). Together both deletions affect 90 genes, including several genes implicated in synaptic function (*DLG4*, *NLGN2*, *CHRNB1*, *GABARAP*, and *PITPNM3*) (Table S2). In AD case RX1107 (age at onset 88), we detected a 2.9 Mb deletion on chromosome 7q35-q36.1 (Figure S2C) that disrupts the *CNTNAP2* gene encoding the contactin-associated protein-like 2 protein, a member of the neurexin family that mediates interactions between neurons and glial cells. SNPs in the *CNTNAP2* were reported to be significantly associated with schizophrenia and bipolar disorder in GWAS studies (Wang *et al*. 2010; O′Dushlaine *et al*. 2011). Intriguingly, variants in *CNTNAP2* were also implicated in pseudoexfoliation syndrome (Krumbiegel *et al*. 2011) among patients who show a selective downregulation of clusterin (CLU) expression in their eyes (Zenkel *et al*. 2006). Notably, the association between *CLU* SNPs and AD was confirmed in several studies at a genome-wide significance level (Harold *et al*. 2009; Lambert *et al*. 2009; Carrasquillo *et al*. 2010).

In addition, we generated a list of 29 genes that were affected by CNVs in two or more AD patients, that were not seen in our Caribbean Hispanic controls, and that were absent or rare in the DGV and 5000 Caucasian controls (Table 3). Some of these genes have potential functional connections to neurological disorders. For instance, in two AD patients, we detected deletions affecting the protein-tyrosine phosphatase receptor-type delta gene (*PTPRD*), which has been associated with restless legs syndrome (Morris *et al*. 2010; Yang *et al*. 2011). One of the deletions (128 kb) removes exon 9 of *PTPRD*, and the other one (135 kb) removes exon 4. Also, two patients (RM4073 and RM4285) had a 622 kb duplication on chr 5q12.1 affecting five genes, including the *NDUFAF2* gene that encodes a chaperone for mitochondrial complex I assembly and that was found to be implicated in attention-deficit/hyperactivity disorder (Lesch *et al*. 2011). Two other duplications were detected at 3p26.3, disrupting the contactin 6 gene (C*NTN6*). Structural and sequence variations in several members of the contactin gene family were associated with neuropsychiatric disorders (*e.g.* schizophrenia and autism) (Fernandez *et al*. 2008; Burbach and van der Zwaag 2009; Cottrell *et al*. 2011).

**Table 3:** Genes affected by CNVs in two or more AD patients

Sample	Chr:start-end	Size	CNVtype	Cytoband	RefSeq genes	Genes overlapped by CNVs in ≥ 2 AD-cases and absent in Caribbean Hispanic controls-without-AD*^a^*	DGV*^b^* (diverse populations)	5000 additional Caucasian-controls*^b^*	#RefSeq genes	Disrupted_genes	Genes with exons_in_cnv	Mouse_MGI_neuronal-phenotypes	Synaptic_genes	Flanked_by_segdups
RM3803	7:16882053- 17397887	515,835	Gain	7p21.1	AHR,AGR3	AHR,AGR3	Rare	Absent	2	AGR3	AHR,AGR3	—	—	—
RX0319	7:16887616- 17381309	493,694	Gain	7p21.1	AHR,AGR3									
RM4226	22:44368703- 44637724	269,022	Gain	22q13.31	ATXN10, FBLN1	ATXN10, FBLN1	Absent	Absent	2	FBLN1	ATXN10,FBLN1	FBLN1	—	—
NY2608	22:44315725- 44638874	323,150	Gain	22q13.31	ATXN10, FBLN1									
RM0949	1:242659903- 242869328	209,426	Gain	1q44	C1orf101, ADSS	C1orf101, ADSS	Absent	Absent	2	C1orf101,ADSS	C1orf101,ADSS	—	—	—
RX1192	1:242659903- 242869328	209,426	Gain	1q44	C1orf101, ADSS									
RM6264	3:643003- 1118424	475,422	Gain	3p26.3	CNTN6	CNTN6	Rare	Rare	1	CNTN6	CNTN6	CNTN6	—	—
NY0553	3:1118424- 1756739	638,316	Gain	3p26.3	CNTN6									
RM5037	16:21907345- 22282331	374,987	Gain	16p12.1	EEF2K, CDR2, POLR3E, C16orf52, PDZD9, VWA3A	EEF2K, CDR2, POLR3E, C16orf52, PDZD9, VWA3A	Rare	Rare	6	CDR2,C16orf65	EEF2K,C16orf65,CDR2,POLR3E,C16orf52,VWA3A	—	—	Yes
RX0490	16:21907345-22282331	374,987	Gain	16p12.1	EEF2K, CDR2, POLR3E, C16orf52, PDZD9, VWA3A									
RM4285	5:60056277-60678352	622,076	Gain	5q12.1	ERCC8, ELOVL7, ZSWIM6, C5orf43, NDUFAF2	ERCC8, ELOVL7, ZSWIM6, C5orf43, NDUFAF2	Absent	Absent	5	ZSWIM6	ERCC8, ELOVL7, ZSWIM6, C5orf43, NDUFAF2	ERCC8	—	—
RM4073	5:60056277-60678352	622,076	Gain	5q12.1	ERCC8, ELOVL7, ZSWIM6, C5orf43, NDUFAF2									
RM5824	3:99076070- 99196494	120,425	Gain	3q11.2	MINA, GABRR3, CRYBG3	MINA,GABRR3,CRYBG3	Absent	Absent	3	GABRR3	MINA,GABRR3,CRYBG3	—	—	—
RX1129	3:99085427- 99196494	111,068	Gain	3q11.2	MINA, GABRR3, CRYBG3									
NY0709	5:80056924- 80293554	236,631	Gain	5q14.1	MSH3, RASGRF2	MSH3,RASGRF2	Absent	Absent	2	MSH3, RASGRF2	MSH3, RASGRF2	RASGRF2	—	—
NY2075	5:80056924- 80293554	236,631	Gain	5q14.1	MSH3,RASGRF2									
RM4073	9:8893857- 9022105	128,249	Loss	9p24.1,9p23	PTPRD	PTPRD	Absent	Absent	1	PTPRD	PTPRD	PTPRD	PTPRD	—
NY2092	9:9850735- 9985938	135,204	Loss	9p23	PTPRD									
NY1942	3:12610706- 12792622	181,917	Gain	3p25.1	RAF1, TMEM40	RAF1,TMEM40	Rare	Rare	2	RAF1	RAF1, TMEM40	RAF1	—	—
RM5553	3:12615745- 12781123	165,379	Gain	3p25.1	RAF1, TMEM40									
RX1208	13:19109434- 19362188	252,755	Gain	13q12.11	MPHOSPH8, PSPC1, ZMYM5	MPHOSPH8	Rare	Rare	3	MPHOSPH8	MPHOSPH8, PSPC1, ZMYM5	—	—	—
NY0295	13:19109434- 19362188	252,755	Gain	13q12.11	MPHOSPH8, PSPC1, ZMYM5									
NY1684	13:19109434- 19362188	252,755	Gain	13q12.11	MPHOSPH8, PSPC1, ZMYM5									

a Controls from the Caribbean Hispanic dataset.

b Controls from databases that were not specifically screened for AD.

## DISCUSSION

We conducted a genome-wide scan for large CNVs (≥100 kb) in a case-control dataset of Caribbean Hispanic origin that was previously investigated in a SNP-based GWAS (Lee JH *et al*. 2010). To generate results with high confidence, we focused on CNVs that were identified by at least two algorithms. We detected 1774 stringent CNVs (Table S4). First, we tested the hypothesis that rare CNVs (≤1%) with a potentially strong impact on AD risk in individual patients might contribute to the overall disease risk, as was previously observed in other common neuropsychiatric disorders (Kirov *et al*. 2009; Zhang *et al*. 2009; Glessner *et al*. 2010). However, the burden analyses of rare CNVs did not find significant differences between cases and controls in CNV rate, total or average CNV size, or the number of genes affected by CNVs.

In addition, we conducted a case-control analysis of large genic CNVs, including common variants, using PLINK regional analysis. The only nominally significant result that survived qPCR confirmation was detected for a duplication on chromosome 15q11.2 affecting up to five genes, including *NIPA1* and *CYFIP1* (*P* = 0.037). Duplications affecting the *NIPA1* and *CYFIP1* in control populations are cataloged at the DGV based on four studies (Pinto *et al*. 2007; Zogopoulos *et al*. 2007; Itsara *et al*. 2009; Shaikh *et al*. 2009) with similar frequencies to our controls (0.5%): this duplication was reported in 24 out of 5056 individuals.

*NIPA1* encodes a magnesium transporter associated with early endosomes in neuronal and epithelial cells (Rainier *et al*. 2003; van der Zwaag *et al*. 2010). CYFIP1 forms a complex at synapses with the fragile X mental retardation protein (FMRP) and eIF4E (FMRP-CYFIP1-eIF4E complex). FMRP acts as an APP translation repressor (Lee EK *et al*. 2010), releasing CYFIP1 from the FMRP-CYFIP1-eIF4E complex in response to synaptic stimulation (Napoli *et al*. 2008). Therefore, unbalanced dosage of CYFIP1 might result in altered APP turnover in AD patients. Of note, this region belongs to a larger region at chromosome 15q11-q13 that has been introduced as one of the most reliable “cytogenetic regions of interest” for genomic aberrations in autism spectrum disorders (Vorstman *et al*. 2006). It is important that the association between AD and the 15q11.2 duplication be validated in follow-up studies using large case-control datasets.

Our study does not support the previously reported marginal association between AD and the ∼500 kb duplication on chromosome 15q13.3 affecting the *CHRNA7* locus in a genome-wide scan of a North American dataset (Heinzen *et al*. 2010), which is 9.5 Mb away from the duplication on 15q11.2 discussed above. We observed an equal number of cases (n = 2; 0.5%) and controls (n = 2; 0.6%) with duplications affecting *CHRNA7*, whereas Heizen *et al.* detected this CNV in six cases (2%) and one control (0.3%) (Heinzen *et al*. 2010).

In addition, a higher copy number of a complex multiallelic segment (DGV variation_0316) containing the olfactory receptor genes on chromosome 14q11.2 was reported to be associated with a decrease in age at onset of AD using genotypes obtained from Affymetrix SNP 6.0 arrays (controls were not evaluated) (Shaw *et al*. 2011). Although this region is ∼200 kb in size, it is poorly covered with SNPs in the 650Y array used for our study (three SNPs). Therefore, we were unable to assess the contribution of this region to AD in our dataset.

We did not detect CNVs (including common variants) that affect the well-confirmed AD loci reported by large GWAS (*CLU*, *PICALM*, *BIN1*, *CR1*, *MS4A4/MS4A6E*, *CD2AP*, *CD33*, *EPHA1*, and *ABCA7*) (Harold *et al*. 2009; Lambert *et al*. 2009; Carrasquillo *et al*. 2010; Hollingworth *et al*. 2011; Naj *et al*. 2011). However, as the array technology used in the current study has limitations in detecting small CNVs, future studies must carefully assess the new AD loci using a qPCR approach to detect small CNVs. For instance, by using multiplex amplicon quantification, a recent study reported that an ∼18 kb CNV in the *CR1* gene is associated with AD risk and could explain the strong association between AD and SNPs at the *CR1* locus detected by GWAS (Brouwers *et al*. 2011).

The limitations of our study are the modest dataset size and the fact that the study was not designed for the comprehensive detection of common CNVs. Several analytical challenges in the detection of common CNVs from SNP-intensity data could lead to a high false negative/positive rate. In general, a case-control setting can only test clusterable common CNVs that are well-tagged by common SNPs and are thus effectively screened by SNP-based GWAS (*e.g.* *CR1* study discussed above (Brouwers *et al*. 2011)). On the other hand, the unclusterable CNVs could be of a multiallelic or complex nature (*e.g.* a small deletion within a large CNV duplication) and can only be accurately genotyped using a combination of custom arrays and deep sequencing. Nevertheless, we observed several reliably detected common CNVs that were included in a case-control analysis of genic CNVs (*e.g.* CNV on 15q11.2). Notably, none of the most significant variations previously detected in our SNP-based Hispanic GWAS (*P* < 10^−5^) (Lee JH *et al*. 2010) tag any of the common CNVs identified in the current study.

In summary, in a stringent genome-wide investigation for the global burden enrichment of large rare CNVs, we didn’t find any significant difference between AD cases and controls. However, this finding may indicate the requirement of larger datasets to identify the enrichment of any of the above-mentioned CNVs. Similarly, confirmation of the biological significance of several large CNVs found only in AD patients requires further assessment in large cohorts, as well as functional studies. Nevertheless, modest datasets, such as reported here, can be useful for identifying rare variants for further validation in follow-up studies.

## Supplementary Material

Supporting Information
