# Supplementary material for: Genome-Wide Survey of Large Rare Copy Number Variants in Alzheimer’s Disease Among Caribbean Hispanics
Source: G3 (Bethesda). 2012 Jan 1;2(1):71–8. doi: 10.1534/g3.111.000869 (PMC3276183; doi:10.1534/g3.111.000869)
Supplement: Supporting Information [file supp_2_1_71__index.html]

Supporting Information 

# Genome-Wide Survey of Large Rare Copy Number Variants in Alzheimer’s Disease Among Caribbean Hispanics

## Supporting Information for Ghani *et al.*, 2012

**Files in this Data Supplement:**

- Supporting Information - Figures S1 and S2 and Tables S1-S4 (PDF, 988 KB)
- Figure S1 - Analyses of a duplication on chromosome 15q11.2 (PDF, 380 KB)
- Figure S2 - The examples of CNVs found only in AD patients but not in normal controls (PDF, 480 KB)
- Table S1 - Chromosomal abnormalities larger than 7.5Mb detected during QC (PDF, 76 KB)
- Table S2 - Rare CNV calls with ≥ 100 Kb in AD cases and controls (.xls, 272 KB)
- Table S3 - CNVs larger than 1 Mb in AD cases and controls (.xls, 40 KB)
- Table S4 - 1774 stringent CNVs with sizes ≥100 Kb passed the QC steps in the 392 cases and 357 controls (mean size = 252,651 bp; median size =176,893 bp) (.xls, 344 KB)
