# Supplementary material for: Genome-Wide Survey of Large Rare Copy Number Variants in Alzheimer’s Disease Among Caribbean Hispanics
Source: G3 (Bethesda). 2012 Jan 1;2(1):71–8. doi: 10.1534/g3.111.000869 (PMC3276183; doi:10.1534/g3.111.000869)
Supplement: Supporting Information [file supp_2.1.71_000869SI.pdf]

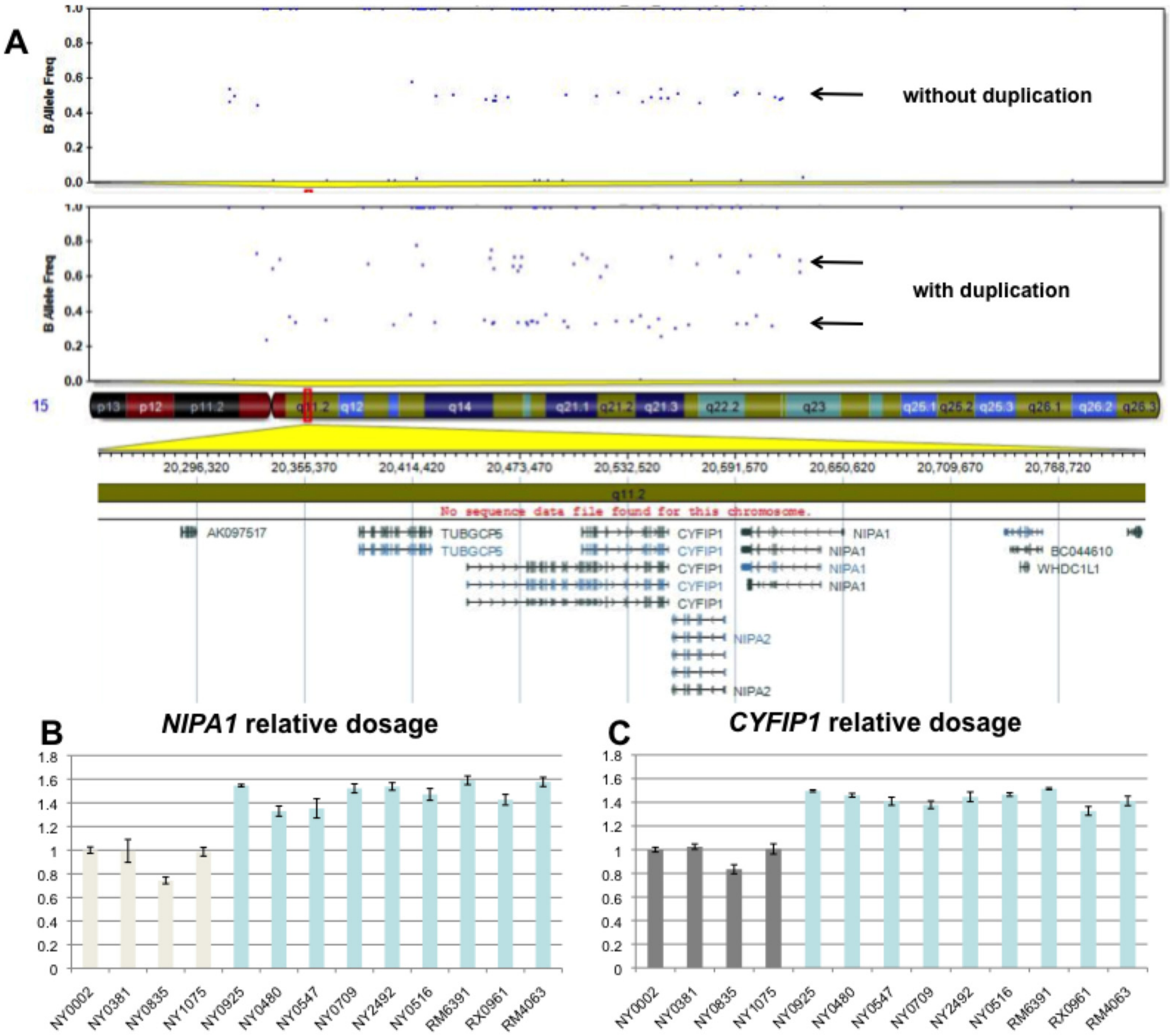

A
without duplication
with duplication
B Allele Freq
B
NIPA1 relative dosage
C
CYFIP1 relative dosage

Figure S1 Analyses of a duplication on chromosome 15q11.2. (A) A split in the B-allele frequency was observed in 10 cases and 3 controls due to a duplication of chromosome 15q11.2. (B) and (C) show the results of quantitative PCR that confirmed the presence of a duplication for the NIPA1 and CYFIP1 loci, respectively. The samples with duplications are highlighted in blue.

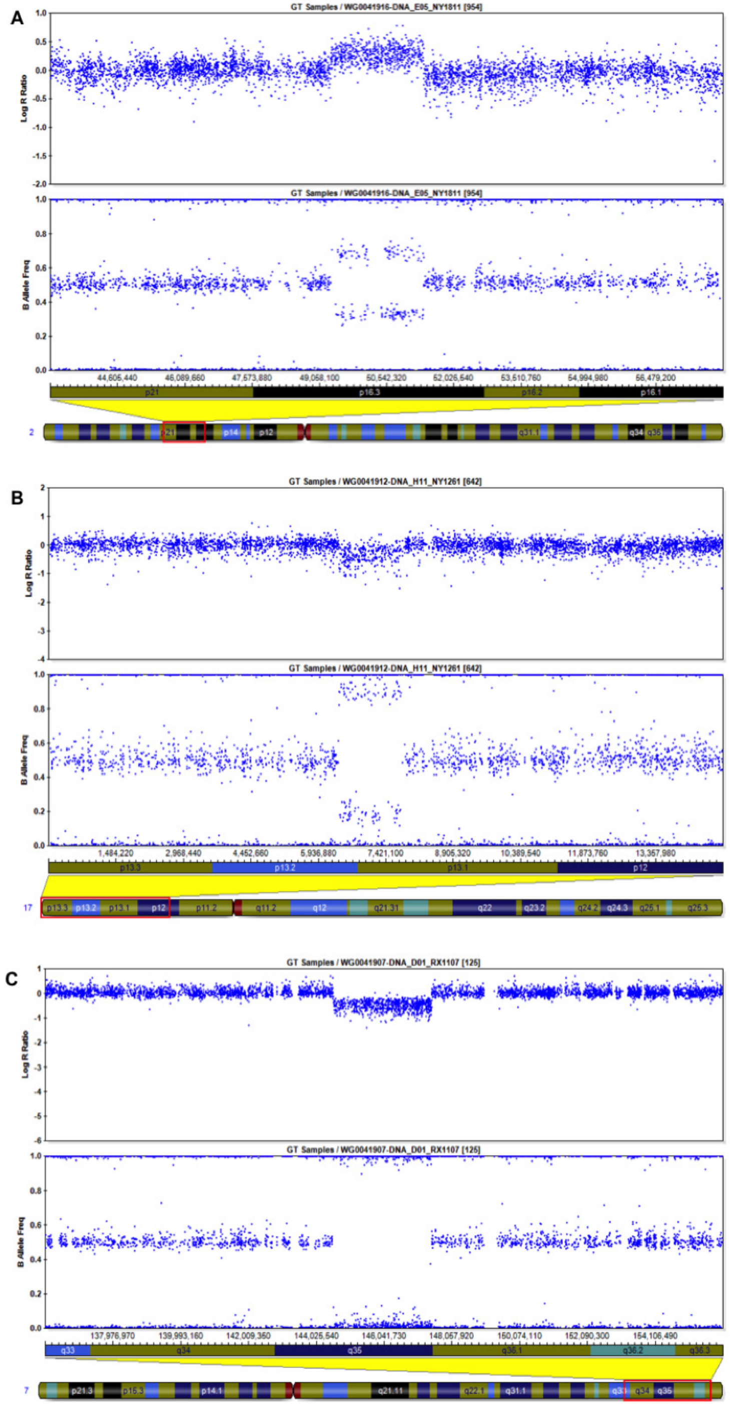
A
GT Samples / WG0041916-DNA_E05_NY1811 [954]
Log R Ratio
B Allele Freq
B
GT Samples / WG0041912-DNA_H11_NY1261 [642]
Log R Ratio
B Allele Freq
C
GT Samples / WG0041907-DNA_D01_RX1107 [125]
Log R Ratio
B Allele Freq

Figure S2 The examples of CNVs found only in AD patients but not in normal controls. (A) Increase in the log R ratio (top) and split in the B-allele frequency (bottom) are consistent with a partial duplication of chromosome 2p16.3. (B) Decrease in the log R ratio (top) and "0 or 1" frequency of the B-allele (bottom) are consistent with a partial deletion of chromosome 17p13.1-p13.2. (C) Decrease in the log R ratio (top) and "0 or 1" frequency of the B-allele (bottom) are consistent with a partial deletion of chromosome 7q35-q36.1.

Table S1 Chromosomal abnormalities larger than 7.5Mb detected during QC.

SampleID	Disease status	Age-at-diagnosis	Gender	chromosome	hg18_chr:start-end	Anomaly type[a]	Comments
NY0243	AD	83	F	1q.arm	whole arm	b.allele.freq.split	mosaicism
NY2512	AD	82	M	4q.arm	whole arm	b.allele.freq.split	mosaicism
NY0790	AD	79	F	4q.arm	whole arm	b.allele.freq.split	mosaicism (almost LOH)
NY1466	Control	*na*	F	4q.arm; 8q.arm	whole arm	b.allele.freq.split	mosaicism
NY1952	AD	75	F	6q22.31-q27; 7q31.1-q33; chr.12	chr6:129Mb-166Mb; chr7:110Mb-141Mb; whole chr.12	6q22.31-q27.low.inten, b.allele.freq.split; 7q31.1-q33.low.inten, b.allele.freq.split; chr12.high.inten, b.allele.freq.split	2 partial deletions (37Mb; 6q22.31-q27) (31Mb; 7q31.1-q33); whole chr.12 duplication-mosaic?
NY0823	AD	68	F	12	whole chr	high.inten, b.allele.freq.split	whole duplication of chr.12
NY0190	AD	65	M	12	whole chr	high.inten, b.allele.freq.split	whole duplication of chr.12
NY1183	AD	90	F	14	whole chr	b.allele.freq.split	mosaicism (almost LOH)
RM4013	Control	88	M	14q31.3-q32.3	chr14:47Mb-106Mb	b.allele.freq.split	mosaicism (almost LOH)
NY1317	AD	76	F	16p.arm	whole p arm; ROH at chr16:56.7Mb-78.2Mb	16p.arm.b.allele.freq.split; (note:there is also a 16q21-q23.1.ROH)	16p.arm.mosaicism
RM1150	AD	87	M	18p.arm	whole arm	b.allele.freq.split	mosaicism
NY0489	AD	67	M	20q11.21-q33	chr20:31Mb-42Mb	low.inten, b.allele.freq.split	partial deletion (11Mb; 20q11.21-q33)
NY1418	AD	85	F	X	whole chr.	b.allele.freq.split	mosaicism

Samples including cases and controls that passed all QC filters and showed CNVs by any algorithm larger than 7.5 Mb on any of the autosomes or chr X were further inspected manually by plotting their log2 ratio intensities as well as allelic genotype ratios. A molecular size cutoff of >7.5 Mb was selected to be consistent with

large cytogenetically visible chromosome abnormalities.

na, not available; ROH,

run of homozygosity

[a]Karyotype information

was not available

Table S2 Rare CNV calls with ≥ 100 Kb in AD cases and controls

Table S2 is available for download at http://www.g3journal.org/lookup/suppl/doi:10.1534/g3.111.000869/-/DC1/TableS2.xls as an excel file.

Table S3 CNVs larger than 1 Mb in AD cases and controls. Eight AD cases and four controls showed CNVs >1Mb. Six such large CNVs were found only in AD cases but were absent in Hispanic controls and DGV.

Table S3 is available for download at http://www.g3journal.org/lookup/suppl/doi:10.1534/g3.111.000869/-/DC1/TableS3.xls as an excel file.

Table S4 1774 stringent CNVs with sizes ≥100 Kb passed the QC steps in the 392 cases and 357 controls (mean size = 252,651 bp; median size =176,893 bp).

Table S4 is available for download at http://www.g3journal.org/lookup/suppl/doi:10.1534/g3.111.000869/-/DC1/TableS4.xls as an excel file.
