# Supplementary material for: Genome-Wide Survey of Large Rare Copy Number Variants in Alzheimer’s Disease Among Caribbean Hispanics
Source: G3 (Bethesda). 2012 Jan 1;2(1):71–8. doi: 10.1534/g3.111.000869 (PMC3276183; doi:10.1534/g3.111.000869)
Supplement: Supporting Information [file supp_2.1.71_FigureS1.pdf]

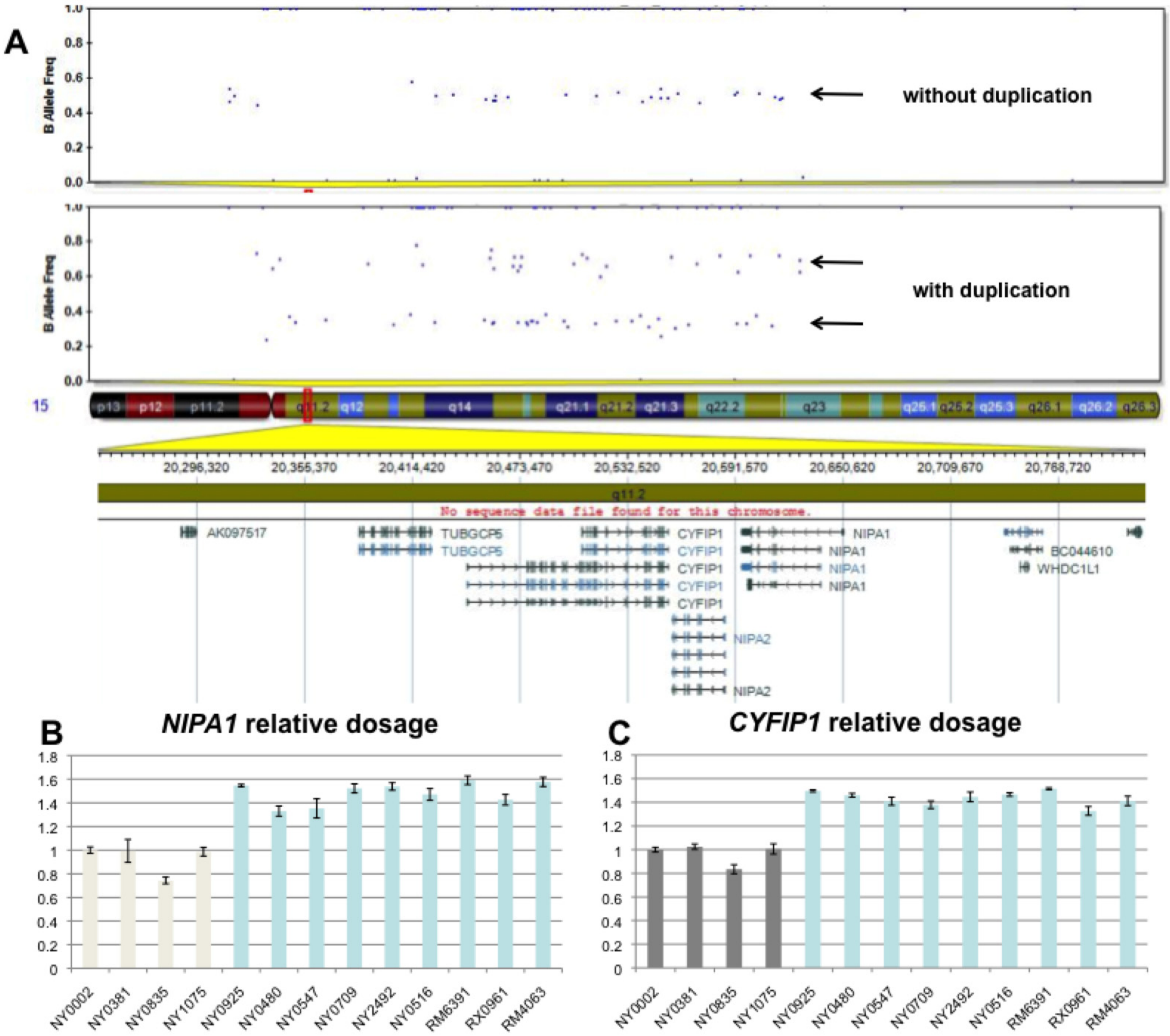
A
B Allele Freq
without duplication
with duplication
15
p13
p12
p11.2
q11.2
q12
q14
q21.1
q21.2
q21.3
q22.2
q23
q25.1
q25.2
q25.3
q26.1
q26.2
q26.3
20,296,320
20,355,370
20,414,420
20,473,470
20,532,520
20,591,570
20,650,620
20,709,670
20,768,720
No sequence data file found for this chromosome.
AK097517
TUBGCP5
CYFIP1
NIPA1
NIPA2
BC044610
WHDC1L1
B
NIPA1 relative dosage
C
CYFIP1 relative dosage
NY0002
NY0381
NY0835
NY1075
NY0925
NY0480
NY0547
NY0709
NY2492
NY0516
RM6391
RX0961
RM4063

Figure S1 Analyses of a duplication on chromosome 15q11.2. (A) A split in the B-allele frequency was observed in 10 cases and 3 controls due to a duplication of chromosome 15q11.2. (B) and (C) show the results of quantitative PCR that confirmed the presence of a duplication for the NIPA1 and CYFIP1 loci, respectively. The samples with duplications are highlighted in blue.
