# Supplementary material for: Genome-Wide Survey of Large Rare Copy Number Variants in Alzheimer’s Disease Among Caribbean Hispanics
Source: G3 (Bethesda). 2012 Jan 1;2(1):71–8. doi: 10.1534/g3.111.000869 (PMC3276183; doi:10.1534/g3.111.000869)
Supplement: Supporting Information [file supp_2.1.71_FigureS2.pdf]

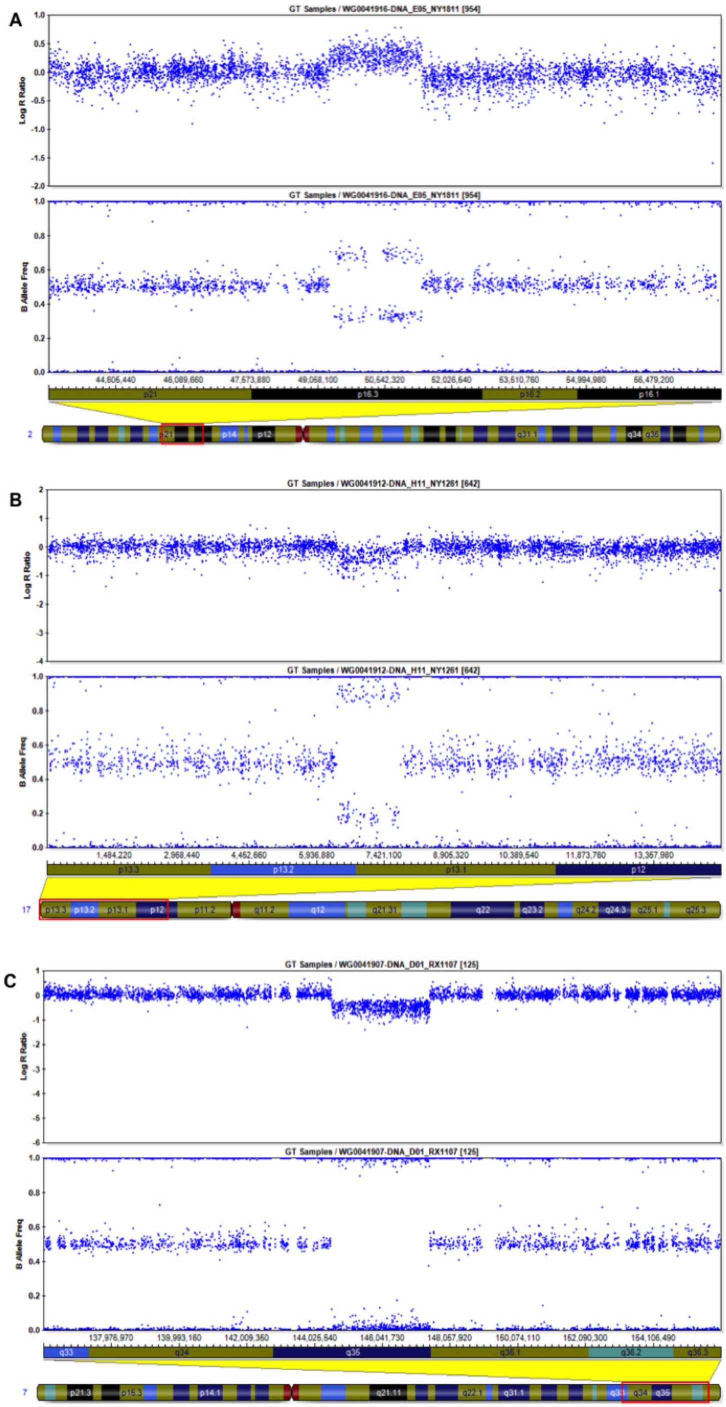
A
GT Samples / WG0041916-DNA_E05_NY1811 [954]
Log R Ratio
B Allele Freq
B
GT Samples / WG0041912-DNA_H11_NY1261 [642]
Log R Ratio
B Allele Freq
C
GT Samples / WG0041907-DNA_D01_RX1107 [125]
Log R Ratio
B Allele Freq

Figure S2 The examples of CNVs found only in AD patients but not in normal controls. (A) Increase in the log R ratio (top) and split in the B-allele frequency (bottom) are consistent with a partial duplication of chromosome 2p16.3. (B) Decrease in the log R ratio (top) and “0 or 1” frequency of the B-allele (bottom) are consistent with a partial deletion of chromosome 17p13.1-p13.2. (C) Decrease in the log R ratio (top) and “0 or 1” frequency of the B-allele (bottom) are consistent with a partial deletion of chromosome 7q35-q36.1.
