# Supplementary material for: Genome-Wide Survey of Large Rare Copy Number Variants in Alzheimer’s Disease Among Caribbean Hispanics
Source: G3 (Bethesda). 2012 Jan 1;2(1):71–8. doi: 10.1534/g3.111.000869 (PMC3276183; doi:10.1534/g3.111.000869)
Supplement: Supporting Information [file supp_2.1.71_TableS1.pdf]

Table S1 Chromosomal abnormalities larger than 7.5Mb detected during QC.

SampleID	Disease status	Age-at-diagnosis	Gender	chromosome	hg18_chr:start-end	Anomaly type[a]	Comments
NY0243	AD	83	F	1q.arm	whole arm	b.allele.freq.split	mosaicism
NY2512	AD	82	M	4q.arm	whole arm	b.allele.freq.split	mosaicism
NY0790	AD	79	F	4q.arm	whole arm	b.allele.freq.split	mosaicism (almost LOH)
NY1466	Control	*na*	F	4q.arm; 8q.arm	whole arm	b.allele.freq.split	mosaicism
NY1952	AD	75	F	6q22.31-q27; 7q31.1-q33; chr.12	chr6:129Mb-166Mb; chr7:110Mb-141Mb; whole chr.12	6q22.31-q27.low.inten, b.allele.freq.split; 7q31.1-q33.low.inten, b.allele.freq.split; chr12.high.inten, b.allele.freq.split	2 partial deletions (37Mb; 6q22.31-q27) (31Mb; 7q31.1-q33); whole chr.12 duplication-mosaic?
NY0823	AD	68	F	12	whole chr	high.inten, b.allele.freq.split	whole duplication of chr.12
NY0190	AD	65	M	12	whole chr	high.inten, b.allele.freq.split	whole duplication of chr.12
NY1183	AD	90	F	14	whole chr	b.allele.freq.split	mosaicism (almost LOH)
RM4013	Control	88	M	14q31.3-q32.3	chr14:47Mb-106Mb	b.allele.freq.split	mosaicism (almost LOH)
NY1317	AD	76	F	16p.arm	whole p arm; ROH at chr16:56.7Mb-78.2Mb	16p.arm.b.allele.freq.split; (note:there is also a 16q21-q23.1.ROH)	16p.arm.mosaicism
RM1150	AD	87	M	18p.arm	whole arm	b.allele.freq.split	mosaicism
NY0489	AD	67	M	20q11.21-q33	chr20:31Mb-42Mb	low.inten, b.allele.freq.split	partial deletion (11Mb; 20q11.21-q33)
NY1418	AD	85	F	X	whole chr.	b.allele.freq.split	mosaicism

Samples including cases and controls that passed all QC filters and showed CNVs by any algorithm larger than 7.5 Mb on any of the autosomes or chr X were further inspected manually by plotting their log2 ratio intensities as well as allelic genotype ratios. A molecular size cutoff of >7.5 Mb was selected to be consistent with

large cytogenetically visible chromosome abnormalities.

na, not available; ROH,
run of homozygosity

[a]Karyotype information
was not available
